# It is time to address the contribution of cholesterol in all apoB-containing lipoproteins to atherosclerotic cardiovascular disease

**DOI:** 10.1093/ehjopen/oeae057

**Published:** 2024-07-29

**Authors:** Peter P Toth, Maciej Banach

**Affiliations:** Department of Preventive Cardiology, CGH Medical Center, 101 East Miller Road, Sterling, IL 61081, USA; Cicarrone Center for the Prevention of Cardiovascular Disease, Johns Hopkins University School of Medicine, Baltimore, MD, USA; Cicarrone Center for the Prevention of Cardiovascular Disease, Johns Hopkins University School of Medicine, Baltimore, MD, USA; Department of Preventive Cardiology and Lipidology, Medical University of Lodz, 93-338 Lodz, Poland

**Keywords:** Apolipoprotein B, Atherosclerosis, Cholesterol, Lipoprotein lipase, Remnant lipoprotein, Residual risk

## Abstract

On average, LDL particles are the most populous lipoprotein in serum under fasting conditions. For many reasons, it has been the primary target of lipid-lowering guidelines around the world. In the past 30 years, we have witnessed remarkable changes in each iteration of dyslipidaemia guidelines, with LDL-cholesterol (LDL-C) targets becoming lower and lower among patients at high and very high risk for atherosclerotic cardiovascular disease (ASCVD). The world over, goal attainment rates are low, and hence, ASCVD prevalence remains unacceptably high. Inadequate LDL-C lowering is a major issue in contemporary cardiovascular (CV) medicine. Another issue that vexes even the most astute clinician is that of ‘residual risk’, meaning the excess risk that remains even after LDL-C is appropriately reduced. In recent years, an important new component of residual risk has emerged: triglyceride-enriched lipoproteins or remnant lipoproteins. These precursors to LDL particles can assume outsized importance among patients with derangements in triglyceride metabolism (e.g. genetic variants, insulin resistance, and diabetes mellitus) and may be more atherogenic than LDL species. Consequently, to reduce total risk for acute CV events, the time has come to include the entire spectrum of apoB-containing lipoproteins in approaches to both risk evaluation and treatment.

## Graphical Abstract

**Graphical Abstract:**
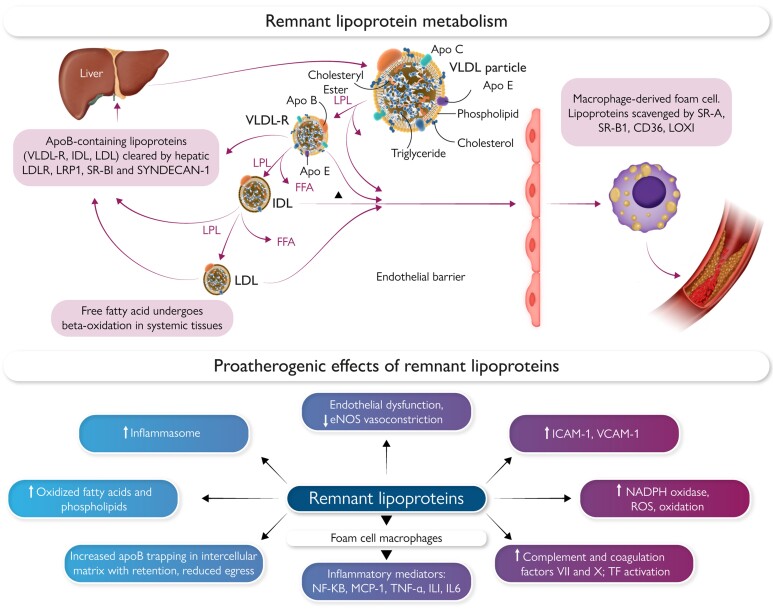
A. Pathways for apolipoprotein B–containing lipoprotein uptake by the liver or scavenging by macrophages in the sub-endothelial space of arteries. In hepatocytes, microsomal triglyceride transferase lipidates apolipoprotein B100 (apoB) to produce very–low-density lipoprotein (VLDL) particles. Very–low-density lipoprotein particles are highly enriched with triglyceride in their core. Once in the central circulation, the VLDL can acquire other apolipoproteins, such as apoE or a variety of C apolipoproteins (apoCII, apoCIII). Within the capillaries of systemic tissues, the VLDL particles undergo delipidation with hydrolysis of triglyceride by lipoprotein lipase, which liberates free fatty acid for (i) oxidative metabolism or (ii) re-assimilation into tissue triglyceride (e.g. in the adipocyte). As the VLDL particle becomes progressively smaller due to the loss of triglyceride mass, it forms VLDL remnants, followed by intermediate-density lipoproteins (IDLs), and then the end-product of lipoprotein metabolism, LDLs. Remnant lipoproteins are cleared by the liver by binding to cell surface receptors such as the LDL receptor (LDLR), the LDL receptor-related protein-1 (LRP-1), scavenger receptor B-I (SR-BI), or syndecan-1 via their apoB or apoE moieties. Alternatively, if hepatic clearance mechanisms are inadequate, then the entire spectrum of apoB-containing lipoproteins can transcytose across the endothelium of arteries and become entrapped within the sub-endothelial space. These particles can subsequently be scavenged by resident macrophages by a variety of cell surface receptors including scavenger receptor A (SRA), cluster of differentiation 36 (CD36), SR-BI, or the oxidized LDL receptor (LOX-1). The scavenged lipoproteins potentiate an inflammatory response, lead to foam cell formation, and the establishment and progression of atherosclerotic disease. B. Pro-atherogenic effects of remnant lipoproteins. Remnant lipoproteins induce endothelial dysfunction and create and more pro-inflammatory, pro-oxidative, and pro-thrombotic environment that fosters atherogenesis. Abbreviations: apoB, apolipoprotein B100; apoC, apolipoprotein C (including apoCI, apoCII, and apoCIII); apoE, apolipoprotein E; eNOS, endothelial nitric oxide synthase; FFA, free fatty acid; ICAM-1, intercellular adhesion molecule-1; IDLR, intermediate-density lipoprotein receptor; IL-1, interleukin-1; IL-6, interleukin-6; LDL, low-density lipoprotein; LDLR, low-density lipoprotein receptor; LOX-1, oxidized LDL receptor-1; LPL, lipoprotein lipase; LRP-1, low-density lipoprotein receptor-related protein-1; MCP-1, monocyte chemoattractant protein-1; NF-KB, nuclear factor kappa-B; ROS, reactive oxygen species (such as superoxide anion and hydroxyl radical); SRA, scavenger receptor A; SR-BI, scavenger receptor BI; TNF-alpha, tumour necrosis factor-alpha; TF, tissue factor; VCAM-1, vascular cell adhesion molecule-1; VLDL, very–low-density lipoprotein.

## Introduction

Atherosclerotic cardiovascular disease (ASCVD) is epidemic throughout the world. Tremendous attention has been devoted to the role of dyslipidaemia in the aetiology of atherosclerosis. Since the introduction of the 3-hydroxy-3-methyl-glutaryl-coenzyme A reductase inhibitors (i.e. statins), the focus of contemporary cardiovascular (CV) medicine has been to reduce LDL cholesterol (LDL-C) to progressively lower levels as more clinical trials have convincingly demonstrated that ‘lower is better and for longer’ and the ‘the earlier the better’ in terms of event rate reduction.^1^ In general, clinicians wait until patients develop ASCVD before they initiate appropriate lipid-lowering therapy. Given this, a critical opportunity to truly intervene in the primary care setting before disease is established is lost. This clinical inertia toward withholding appropriate therapy must be overcome. However, as shown in the Further Cardiovascular Outcomes Research with PCSK9 Inhibition in Subjects with Elevated Risk (FOURIER) trial, even after achieving a median LDL-C level of 0.72 mmol/L (30 mg/dL) at 48 weeks, combination therapy with a statin and a proprotein convertase subtilisin-kexin type 9 (PCSK9) inhibitor provided only a 15% incremental reduction in the primary composite of major acute coronary events (MACE; 5 points) over statin monotherapy with 9.8% of patients still experiencing a MACE event over a median of 2.2 years of follow-up.^2^ Clearly, even with very low attained LDL-C, there remains significant residual risk for MACE.^3^ To date, no prospective clinical trials have been able to demonstrate that either raising HDLs or reducing triglycerides (TGs) impacts risk for ASCVD-related events, such as myocardial infarction (MI), ischaemic stroke, or CV mortality.

## Residual risk

Residual risk for MACE is clearly not just a matter of aggressive LDL-C reduction. Inadequately controlled hypertension, diabetes mellitus, smoking, inflammation, lipoprotein(a) [Lp(a)], sedentary lifestyle, and social and psychiatric stress (among others) all play a role. In the Clinical Outcomes Utilizing Revascularization and Aggressive Drug Evaluation (COURAGE) trial, risk factor background was relatively well treated even by contemporary standards, and participants had high rates of adherence to dietary intervention, regular exercise, and smoking cessation. Despite this, the participants treated with optimal medical therapy still experienced a component of the primary composite endpoint (MI, ischaemic stroke, or death) of 19.5% over a median of 4.6 years of follow-up.^4^ Is there a limit to risk reduction once atherosclerotic disease has become established? It is premature to make such an admission, and it would constitute a form of therapeutic nihilism, which we find difficult to accept. Clearly, reducing the intensity of inflammation with colchicine has been shown to correlate with incremental reduction in MACE rates in patients with ASCVD.^5^ Do other apoB-containing lipoproteins besides Lp(a) correlate with risk and should they be treated?

## Lipoprotein metabolism

Very–low-density lipoprotein is highly triglyceride-enriched and serves to distribute fatty acid to systemic tissues to drive oxidative metabolism. Triglycerides are carried within the core of the lipoprotein particle and are hydrolysed by the enzyme lipoprotein lipase (LPL). The regulation of LPL activity is complex.^6^ Known activators of this enzyme include apoCII, apoE, apoA5, and fibroblast growth factor 21 (FGF-21); inhibitors include apoCIII and angiopoietin-like proteins (ANGPTL) 3 and 4. In the setting of insulin resistance and diabetes, LPL is inhibited due to high levels of apoCIII and reduced levels of apoCII. The inhibition of LPL or genetic deficiency in enzyme activity correlates with increased serum levels of triglycerides, triglyceride-enriched lipoproteins (TRLs) or ‘remnants’, and heightened risk for ASCVD.^7^ Very–low-density lipoprotein is lipolysed to progressively smaller lipoproteins due to reductions in triglyceride mass, yielding in sequence: VLDL remnants of different sizes, intermediate-density lipoprotein (IDL), and then LDL, the end-product of VLDL metabolism (*Graphical Abstract*). As the particles become smaller, they become more concentrated with cholesterol ester. Very–low-density lipoprotein, VLDL remnants, IDL, and LDL all contain apoB. Elevated serum levels of apoB reflect high levels of total atherogenic lipoprotein burden in serum and heightened risk for ASCVD.^8,9^

## Remnant lipoproteins

In general, total remnants are considered to be the sum of VLDL remnants and IDL. The idea that TRLs were, in addition to LDL, atherogenic was proposed by Zilversmit in 1979. Since then, multiple prospective longitudinal cohorts have demonstrated a strong, significant association between lipoprotein remnant particles and risk for ASCVD in both men and women and people of different racial and ethnic backgrounds.^10–14^ For example, in the Atherosclerosis Risk in Communities (ARIC) study, remnant lipoprotein cholesterol (RLP-C; estimated as non–HDL-C–LDL-C) had a stronger association with ASCVD than did LDL-C even after comprehensive adjustment for risk covariates [hazard ratios (HRs) 1.59 (95% CI: 1.39–1.82) vs. 1.03 (0.77–1.37)]. In the Women’s Health Study, RLPs were associated with the following statistically significant HRs for specific cardiovascular endpoints: total CVD, 1.87 (1.14–3.06); MI, 3.05 (1.46–6.39); peripheral arterial disease, 2.58 (1.18–5.63); and CV and cerebrovascular events, 1.79 (1.04–3.07). In a meta-analysis of the Jackson Heart Study and the Framingham Offspring Cohort Study, for each standard deviation increase in RLP-C, the risk for new-onset ischaemic heart disease increased significantly (HR 1.23; 95% CI 1.06–1.47).^11^ In the Copenhagen General Population Study, elevated RLP-C was associated with a marked increased risk for peripheral arterial disease (HR 4.8; 95% CI 3.1–7.5).^15^ Several recent meta-analyses also corroborate the important impact of RLPs on risk for ASCVD-related events.^16,17^ A Mendelian randomization study that included just under 1 000 000 persons showed that RLP-C increased risk for coronary artery disease, MI, and ischaemic stroke.^18^ The inclusion of RLPs in risk prediction also improves identification of those at heightened risk for MI and development of ischaemic heart disease.^19^

Is it biologically plausible that RLPs are so atherogenic? Yes. The RLPs exert a broad spectrum of pro-atherogenic phenomena (*Graphical Abstract*) and are more pro-inflammatory than LDL.^20,21^ In addition, unlike LDL, which has to be oxidatively modified before it is scavenged by macrophages, RLPs do not require any form of chemical modification in order to be recognized and cleared by macrophages.^22^ Because RLPs are larger than LDL particles, they also have the capacity to deliver more cholesterol into atherosclerotic lesions per particle than an LDL particle.

The Progression of Early Subclinical Atherosclerosis (PESA) study has shown that even quite modest elevations in serum triglycerides (1.13 mmol/L or 100 mg/dL) correlate with increased risk for coronary and peripheral arterial atheroscleroses as well as heightened arterial wall inflammation.^23^ Are the triglycerides bad actors, or do they reflect increased levels of triglyceride-enriched VLDL and RLP particles in the central circulation? Triglycerides do not accumulate in atherosclerotic plaque. Cholesterol does. Somatic cells oxidize triglycerides and their constituent fatty acids in the mitochondrial matrix. Somatic cells do not have the capacity to catabolize cholesterol.

As shown by the Copenhagen Heart Study investigators, VLDL cholesterol and IDL + LDL cholesterol account for 50% and 29% of the risk, respectively, for MI after multivariable adjustment for risk covariates.^24^ Very–low-density lipoprotein triglyceride contributed another 8%, possibly because it is a source of saturated long-chain fatty acids, which can be oxidized in the sub-endothelial space and promote an inflammatory response. Overwhelmingly, it is the cholesterol in apoB-containing lipoproteins that drives atherosclerotic plaque development and progression. Because triglyceride must be incorporated into lipoproteins in order to be miscible and transported in plasma, it is likely simply functioning as a biomarker reflecting elevated levels of VLDL and remnant particles.

The relationships between VLDL particles and its remnants with atherogenesis is complex. In a Mendelian randomization study from Copenhagen that included 15 genetic variants and evaluated risk for developing ischaemic heart disease, the causal odds ratio (OR) for a 1 mmol/L genetic rise in non-fasting RLP-C was 2.8 (95% CI 1.9–4.2), while the OR for a 1 mmol/L increase in LDL-C was 1.5 (95% CI 1.4–1.7).^22^ In a Mendelian randomization study using the UK Biobank, single-nucleotide polymorphisms associated with RLP-C and LDL-C were investigated; for every 1 mmol/L increase in RLP-C or LDL-C, risk for coronary heart disease was associated with ORs of 2.59 (95% CI 1.99–3.36) and 1.37 (95% CI 1.27–1.48), respectively. In another analysis from the Copenhagen General Population Study, the HRs for myocardial infarction when followed over 10 years were 3.5 and 1.3 for VLDL and the combination of IDL and LDL particles, respectively. These findings all demonstrate that LDL precursors are more atherogenic than LDL particles, underlining the importance of reducing all apoB-containing lipoprotein species. Taken collectively, these studies suggest that RLP-C is approximately twice as atherogenic as LDL-C. Measurements and treatment targets for RLPs would have to take this into consideration and treat accordingly. For those clinicians with access to various technologies/separation methods for measuring RLPs, this information is empowering and makes for a much more refined estimation of lipoprotein-related risk. However, most clinicians do not have such access and a simpler approach can be advocated for.

In this discussion, we have avoided another member of the apoB family, namely, Lp(a). The reason for this is because currently available therapies do not reduce it significantly and clinical trials are underway with novel agents to establish whether or not Lp(a) reduction is efficacious.^25^

## Intensification of treatment

It is established that lowering apoB is effective and that it is a better marker of risk than is LDL or non–HDL-C.^26^ Measurements of apoB include the entire spectrum of lipoproteins from parent VLDL particles to LDLs. Among patients with hypertriglyceridaemia guidelines advocated treating risk-stratified levels of apoB or non–HDL-C.^27^ Debates surrounding apoB have been long-standing and persistent, and we also do not address issues of concordance and discordance.^28,29^ This is for the sake of simplicity. Most clinicians do not read guidelines, and it is highly established that the large majority of high-risk patients are under-treated.^30^ Treating apoB precludes the need to perform costly specialized measurements using a variety of technologies for separating specific RLP fractions, and there is no agreed-upon gold standard for measuring these lipoprotein species.^31^ ApoB is highly validated, the molecular basis for its role in atherogenesis and risk prediction well understood, and it is inexpensive to measure.^32^ In its 2019 guideline, the European Atherosclerosis Society/European Society of Cardiology determined that apoB was a more accurate marker of risk than either non–HDL-C or LDL-C.^33^ It also issued risk-stratified targets for this biomarker (*Table 1*). On the other hand, RLP-C levels can be easily estimated (RLP-C = non–HDL-C – LDL-C), and some clinicians may prefer to assess residual risk and refine risk estimation after LDL-C reduction by calculating RLP-C. It is important to point out that estimated RLP-C is significantly less accurate that measured RLP-C.^34^ Moreover, if it is based on an LDL-C calculated by the Friedewald equation, then it essentially equates to an estimate of serum triglycerides, not RLP-C. Like the addition of non–HDL-C to the standard lipid profile, it would be relatively easy to automatically calculate and incurs no added expense to include estimated RLP-C. Cut points for what constitutes an abnormal RLP-C could then be derived for different populations and adjuvant therapy initiated as indicated.

**Table 1:** Advantages of apoB, non–HDL cholesterol, and remnant lipoprotein cholesterol as biomarkers of cardiovascular risk

Advantages of apolipoprotein B (apoB)	Advantages of non–HDL cholesterol	Advantages of remnant lipoprotein cholesterol
Based on multiple prospective observational studies and discordance analyses, apoB is a more accurate marker of ASCVD risk than LDL-C or non–HDL-C. Hence, apoB is a better risk analyte to select patients for preventive therapy than non–HDL-C	Easy to calculate (total cholesterol–HDL-C) and generally appears in laboratory results	Easy to calculate (total cholesterol–HDL-C–LDL-C) but does not appear as a component of laboratory results. Calculated estimates of RLP-C are also lower and less accurately predictive of CVD risk than measured levels of RLP-C. This calculation has the drawback of including VLDL, a lipoprotein that is not a true remnant
Based on multiple meta-analyses and recent randomized controlled trials of intensive lipid-lowering therapy, it has been demonstrated that apoB is a more accurate marker of residual risk than non–HDL-C in patients on lipid-lowering therapy. apoB, therefore, is a better tool to determine if therapy needs to be intensified whether by increasing statin dose or adding therapies such as ezetimibe or PCSK9 inhibitors	Better predictor of risk than LDL-C, especially in the setting of hypertriglyceridaemia. In patients with elevated serum triglycerides, triglyceride-enriched lipoprotein levels [VLDL and remnant lipoproteins (VLDL remnants and intermediate-density lipoproteins)] can be markedly elevated, leading to significant underestimations of CVD risk	Makes diagnosis of various dyslipidaemias and dysbetalipoproteinaemia (type III dyslipoproteinaemia) straightforward
apoB is a measure of the entire spectrum of atherogenic lipoproteins, from VLDL to Lp(a). Because apoB is a more accurate marker of cardiovascular risk than non–HDL-C, apoB is a more accurate marker of benefit from therapy than non–HDL-C	Better predictor of risk than LDL-C in the non-fasting state. In the non-fasting state, triglyceride levels strongly influence calculated LDL-C when using the Friedewald equation	Like apoB and non–HDL-C, RLP-C represent a significant marker of residual risk and identifies patients who require either greater LDL-C reduction or greater triglyceride reduction
apoB can be quantitated with high accuracy. There is no uniform definition of remnant lipoproteins and many methods (ultracentrifugation, nuclear magnetic resonance, chromatography, antibody-based assays, etc.) have been used to quantify them with no current gold standard having been established, making comparisons among studies challenging	Physicians have greater familiarity with non–HDL-C than apoB, but also less familiarity with non–HDL-C when compared with LDL-C	Disadvantage of RLP-C: Measurement of RLP-C requires the use of separation methods (ultracentrifugation, nuclear magnetic resonance spectroscopy, antibody-based assay, etc.) that can incur significant cost and are at present largely investigational. There is no uniform definition of different remnant lipoprotein fractions between technologies and there is, as yet, no ‘gold standard’ approach to measurement due to extant validation issues
On follow up of patients on therapy, only apoB needs to be measured. Acting on a single number (apoB) is simpler than attempting to interpret five (total cholesterol, triglyceride, non–-HDL-C, LDL-C, and HDL-C). A simpler process of care is a more effective and efficient process of care	Non–HDL-C is also an estimate of total atherogenic lipoprotein burden in serum and a demonstrably better marker of risk than LDL-C	Disadvantage of RLP-C: VLDL-C, VLDL-C remnants, and IDL-C fractions require scaling and use of multipliers since they have hazard ratios greater than that for LDL-C; this may incur more complexity than most physicians will be willing to engage. Risk-stratified goals not yet defined based on accepted standard
apoB must be measured in addition to a standard lipid panel in order to diagnose Fredrickson–Levy-type dyslipidaemias. A non–HDL-C measurement is not sufficient	Misconception: non–HDL-C is not free. It can only be calculated from a standard lipid panel	
Misconception: the measurement of apoB is expensive. It is not	Risk-stratified goals are defined by the European Guidelines for moderate, high, and very–high risk, non–HDL-C should be reduced t <3.4, <2.6, and <2.2 mmol/L	
The measurement of apoB is available throughout the world, and its assay is validated		
Risk-stratified goals are defined by the European Guidelines: for moderate, high, and very–high risk, apoB should be reduced to <1.0, <0.8, and < 0.65 g/L		

There are appropriate adjuvant therapies to select from in order to reduce residual risk. If the residual risk primarily arises from inadequate LDL-C reduction with a statin, then the addition of such agents as ezetimibe, a PCSK9 monoclonal antibody, or bempedoic acid is efficacious for incremental LDL-C reduction. If the statin dosing is low, it can be intensified. In the setting of hypertriglyceridaemia, if the patient is insulin resistant or overweight/obese, aerobic exercise and caloric restriction correlate highly with weight loss, reduction in insulin resistance, and lowering of triglycerides and RLPs.^35–37^ With the advent of the glucagon-like 1 receptor agonists, obesity with or without diabetes can be treated with clear evidence for reductions in acute cardiovascular events, significant weight loss, and improvement in glycaemic indices.^38,39^ Based on a variety of studies using fenofibrate^40^ and pemafibrate,^41^ fibrate therapy does not impact risk for ASCVD-related events. The REDUCE-IT trial showed strong event rate reductions in high-risk patients with hypertriglyceridaemia already on a statin with well-controlled LDL-C treated with icosapent ethyl.^42^ Studies combining emerging therapies for triglyceride reduction (*Table 2*) with established LDL-C–lowering agents will help to further refine our therapeutic interventions for residual risk.

**Table 2:** Emerging therapies that impact serum triglycerides, apoB, and remnant lipoprotein levels

Pharmacologic agent	Mechanism of action	Lipid and lipoprotein modifications
Evinacumab	Monoclonal antibody that neutralizes ANGPTL3	↓TG, apoB, non–HDL-C, LDL-C, apoCIII, as well as VLDL-C and medium and small VLDL-C
Icosapent ethyl	Omega-3 fish oil that stimulates lipoprotein lipase, among other effects. Lipid effects noted are from the ANCHOR trial	↓TG, apoB, non–HDL-C, hsCRP, apoCIII, VLDL- C, VLDL-TG, RLP-C, and oxLDL-C
Lomitapide	Microsomal triglyceride transfer protein inhibitor that reduces the synthesis and secretion of VLDL-C	↓TG, VLDL-C, RLP-C, apoB, non–HDL-C, and LDL-C
Olezarsen	Antisense oligonucleotide that reduces the translation of hepatic apoCIII mRNA	↓TG, apoB, non–HDL-C, and apoCIII; neutral on LDL-C
Pemafibrate	Selective PPAR-_α_ modulator. Lipid effects noted are from PROMINENT trial	↓TG, apoB, VLDL-C, and RLP-C; however, apoB increased
Volanesorsen	Antisense oligonucleotide that reduces the translation of hepatic apoCIII mRNA	↓Chylomicrons, apoB48, VLDL-C, and non–HDL- C; however, apoB and LDL-C increased
Vupanorsen	Antisense oligonucleotide that reduces the translation of hepatic ANGPTL3 mRNA	↓TG, apoB, non–HDL-C, VLDL-C, RLP-C, and LDL-C

ANGPTL3, angiopoietin-like protein 3; apoB, apoB100; hsCRP, high sensitivity C-reactive protein; LDL-C, low-density lipoprotein cholesterol; PPAR, peroxisomal proliferator activated receptor; RLP-C, remnant lipoprotein cholesterol; TG, triglyceride; VLDL-C, very–low-density lipoprotein cholesterol.

We should act now; too many patients on treatment sustain CVD events. The residual risk gap needs to be urgently closed, and no potential opportunity to diminish events should be lost.

## Data Availability

No new data were generated or analysed in support of this research.
